# Basic research at the epicenter of an epidemic

**DOI:** 10.7554/eLife.00639

**Published:** 2013-04-02

**Authors:** William R Bishai

**Affiliations:** K-RITH, Durban, South Africawilliam.bishai@k-rith.org

**Keywords:** TB, HIV, K-RITH, drug resistance, epidemic, South Africa

## Abstract

**William R Bishai**, director of the KwaZulu-Natal Research Institute for Tuberculosis and HIV (K-RITH), argues that the best place to carry out research into a disease is in its midst.

Smallpox may have been eradicated 35 years ago, but we are still battling many other major global health scourges. Malaria, for example, kills some 1.2 million people every year (Murray et al., 2012), and recent cholera epidemics in Zimbabwe, Somalia and Haiti have killed thousands. The death toll from HIV/AIDS is even higher, with almost 2 million deaths last year, but new drugs and health care delivery mechanisms mean that this number is falling.

But there's one major disease where the battle is still being badly lost—tuberculosis. The *Mycobacterium tuberculosis* that felled Frederic Chopin, John Keats, Doc Holliday and countless others—before being largely eliminated in the industrialized world—still kills nearly 2 million people in the developing world every year. Even more alarmingly, in 2005 researchers in the province of KwaZulu-Natal in South Africa spotted an outbreak of a strain of *M. tuberculosis* that was resistant to the four key classes of drugs used to treat the disease. These super drug-resistant bugs have now been found in 58 countries, their spread fuelled by the lethal combination of HIV and TB. The mortality rate worldwide for the victims of this extensively drug resistant TB (XDR-TB) is more than 80%, making diagnosis almost a death sentence.

The scientific and medical challenges are huge. We don't have an effective vaccine. We lack diagnostics and biomarkers. The current drug regimen—multiple drugs that must be taken reliably for six months—is virtually impossible to administer successfully in the developing world, where rural farmers may have to walk three hours to the nearest clinic. Too often, treatment simply leads to the development of drug resistance. And we have only just begun to study the bacterium's biology and to learn how it manipulates the human immune system.

## A new approach

To understand TB and its deadly synergy with HIV, and to develop new diagnostics and treatments, we need more research. But the current research model could use some help. In this article I will describe a new approach that involves bringing world-leading basic research to the epicenter of epidemics, rather than trying to fight diseases from laboratories at universities or government agencies thousands of miles away.

Building state-of-the art basic research facilities at sites where epidemics are raging brings a number of crucial benefits. Most importantly, it takes maximum advantage of the extensive knowledge and skills of those already engaged in fighting the disease. If I had TB, I would opt to be cared for by the clinicians in KwaZulu-Natal. Those doctors and nurses are part of a vibrant well-trained infectious disease community and they treat thousands of cases a year, as opposed to the thirty or so we see annually in Baltimore.

**Figure 1:**
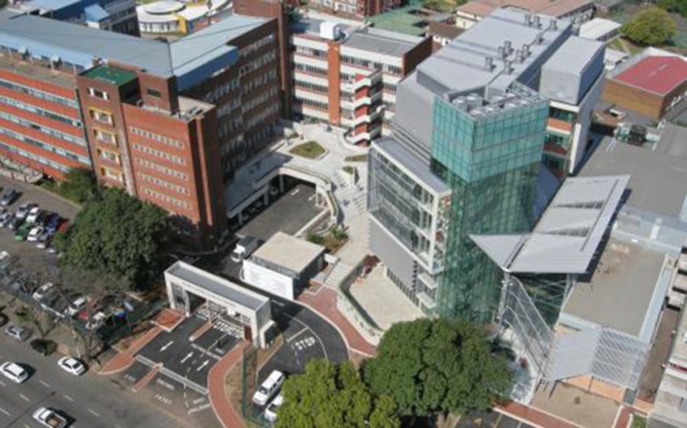
An aerial view of K-RITH.

Moreover, the science will benefit from this approach. To learn whether—and why—some TB strains grow faster than others, to correlate genetic variations with clinical outcomes, and to figure out the mechanisms behind drug resistance, will require access to scores of fresh sputum samples daily. It will require bringing hundreds of patients into the clinic for advanced imaging. It will require tapping into the ideas of those already on the front lines, and putting the most advanced scientific instruments in their hands. It will require lots of money. And transplanting basic scientists from their lives in the West into the heart of an epidemic creates a visceral connection and sense of urgency that is impossible to achieve any other way.

Even more progress will come from training and nurturing homegrown talent, creating a career pathway for motivated students to eventually run their own basic research labs and lead the fight against global diseases. That not only increases the chances of victories against major scourges like TB, it also creates a more equitable scientific community and research culture. The current model of basic science at a distance has raised thorny issues of ‘scientific colonialism’ or ‘parachute science’, where Western researchers have been accused of using knowledge and samples from those working in the field to make the big discoveries and to advance their own careers, leaving local scientists and clinicians out of the loop and out of the limelight.

The idea of building research capabilities in the developing world is not a new one, of course. The Wellcome Trust, for example, has set up five ‘major overseas programmes’ in Africa and Southeast Asia (Wellcome Trust). Those programs have been very successful. They are behind the introduction of artemisinin combination therapy for malaria, now the frontline treatment. They have also proved the worth of insecticide-treated bed nets in reducing malaria infection rates, and have tested promising vaccines. In the TB field, work at the University of Cape Town has highlighted the role of crowding and poor living conditions in the spread of TB in South Africa (Andrews et al., 2013).

The Medical Research Council's initiative in The Gambia, established in 1947, focuses on combating infectious diseases that affect the entire continent, and their Ugandan AIDS unit, integrated with the Uganda Virus Research Institute, is a prime example of leveraging international collaboration to build research capacity in the developing world (Medical Research Council). Such collaborations have been heralded by the Institute of Medicine as important for the global fight against HIV/AIDS (Institute of Medicine, 2010). Started in 1998, the Centre for Global Health Research's Million Death Study in India is currently monitoring nearly 14 million people in one of the world's largest studies of premature mortality. The project includes six working groups that are investigating the global issue of deaths without a certified cause, a phenomenon largely concentrated in low- and middle-income countries (Centre for Global Health Research). By design, however, all of these centres are largely focused on clinical research.

So could we make even more progress by building up basic research capacity at the epicenter of epidemics? It is an idea worth trying. Remember, for instance, the urgent and powerful basic research engine in the United States and Europe that developed treatments for HIV as the AIDS epidemic raged right outside the laboratory door. We could create the same compelling and effective scientific effort in the heart of other epidemics around the world, not just TB and HIV/AIDS, but also air pollution in crowded cities such as Cairo or Bangkok, or the regional cardiomyopathies that are prevalent in some developing countries. The combination of basic research and intensive clinical work in the same place is powerful, as new findings can immediately be explored at the lab bench or put into practice in the field, accelerating both the pace of discovery and the use of new diagnostics, treatments and vaccines.

## Creating K-RITH

I am not the only one who believes in this new approach to addressing global health problems. In the mid-2000s, the Howard Hughes Medical Institute (HHMI) was supporting about 400 investigators in the United States and building its own research campus at Janelia Farm in Virginia. However, under then president Thomas Cech, HHMI decided that it should also provide more support for basic biomedical science elsewhere in the world.

The need for such action was highlighted by the emergence, in 2005–2006, of the super resistant (XDR) strain of *M. tuberculosis* in the remote South African town of Tugela Ferry, where up to 40% of adults are infected with HIV and rates of tuberculosis are 1200 cases per 100,000 people. The larger KwaZulu-Natal province already had one of the highest rates of TB in the world, with 120,000 new cases per year.

Four prominent scientists—William Jacobs Jr (Albert Einstein College of Medicine), Bruce Walker (Massachusetts General Hospital), and Salim S. Abdool Karim and Adriaan Willem Sturm (both of the Nelson R. Mandela School of Medicine in Durban)—decided that something had to be done. They approached Cech and Malegapuru William Makgoba, vice chancellor and principal of the University of KwaZulu-Natal (UKZN), and began discussions. The conclusion: the best hope for quashing the epidemic was to bring more world-class basic research to South Africa. HHMI agreed to provide $75 million to build a new research centre on the campus of the Mandela School of Medicine at UKZN, and to support its operations through to 2018. The centre, called the KwaZulu-Natal Research Institute for Tuberculosis and HIV (K-RITH), would then become independent under South African leadership.

I became K-RITH's first permanent director in 2010. When I took the job, I had two fears. One was that I would be a director without a building. The other was that I would be a director of an empty building. Scientists, naturally, are ambitious. There is a tried and true scientific career path in Europe and North America, and convincing someone to step off this established path for a potentially risky new venture might, I feared, be challenging.

I need not have worried. Construction began in 2010 and was completed only a few weeks behind schedule in September 2012. The institute has five core facilities—microbiology, immunology, pharmacology, high-throughput biology and clinical research services—along with 6190 square feet of biosafety level 3 lab space (for safely handling TB and HIV) and state-of-the-art instruments.

We were also able to recruit truly exceptional scientists, who will each run one of eight independent labs at K-RITH. Three were born in Africa, though trained in the United States, and the other five recruits so far (including me) have come from the United States, Great Britain and France. We would like to have recruited more African scientists from South Africa's leading universities but, as often happens with brand new institutions, it is a challenge to persuade leading researchers to give up productive labs at established universities to build up new labs from scratch somewhere else.

Our scientists have already published more than six dozen papers on the research they will continue at K-RITH. Those papers include an analysis by K-RITH investigator, Alexander Pym, and colleagues of the spread of resistant TB around the world from Tugela Ferry (Cooke et al., 2011), a comparison of treatment outcomes in different parts of KwaZulu-Natal (Loveday et al., 2012), and work by another K-RITH investigator, Adrie Steyn and co-workers on the relationship between iron-containing proteins and the virulence of *M. tuberculosis* (Saini et al., 2012). Steyn's research is part of a larger effort aimed at understanding how the bacterium fends off oxidative attacks from the immune system for many years. In my own lab, we have been able to show that when the bugs infect immune cells called macrophages, they trick the cells into creating a hyperinflammatory response. This brings more immune cells to the site for the bacteria to infect, and causes tissue damage, providing the bacteria with a place to hide (Agarwal et al., 2009).

## Pressing scientific and medical problems

Since its official opening on October 9 last year, K-RITH has been ramping up to its full capacity, tackling a number of fundamental scientific questions. How have some TB variants acquired resistance to virtually all drugs, as seen in the roughly 300 new drug-resistant cases that emerge each year in KwaZulu-Natal province? How do both TB and HIV create disease reservoirs that can lurk for years before rekindling deadly infections? And how can we manipulate the response of the immune system to better thwart the TB and HIV pathogens? Right now, TB and HIV seem to know more about human biology than scientists do.

Right now, TB and HIV seem to know more about human biology than scientists do.

Just as important, K-RITH will address the two most pressing problems in the fight against TB—the lack of good, quick diagnostics and biomarkers, and the dearth of new drugs and treatments. The mainstay TB diagnostic is still the basic smear microscopy technique used by Robert Koch to discover *M. tuberculosis* in 1882: get patients to cough up a sputum sample, stain it with dye, wash it with acid alcohol and put it under a microscope to look for TB bacteria. However, the test spots only about half of those with active disease, and the failure rate is even higher in those with HIV, who often have little disease in their lungs. Most of the patients missed by the sputum test can be diagnosed with a culture test, but because *M. tuberculosis* grows so slowly, a culture takes 10 to 60 days. Many patients from Tugela Ferry with XDR-TB died before they were even diagnosed. And neither the sputum nor the first-line culture tests can determine whether TB strains are resistant to some or all drugs.

There has been some progress in developing better diagnostics. The GeneXpert system developed by Cepheid, Inc. and the Foundation for Innovative New Diagnostics, uses new technology to simultaneously detect DNA sequences specific both to TB and to resistance to the drug rifampicin, and has been hailed as a potential game-changer (Evans, 2011). But the test is relatively expensive, misses a fraction of infected smear-negative TB patients, and tells us nothing about resistance to the nine other commonly used drug classes.

**Figure 2:**
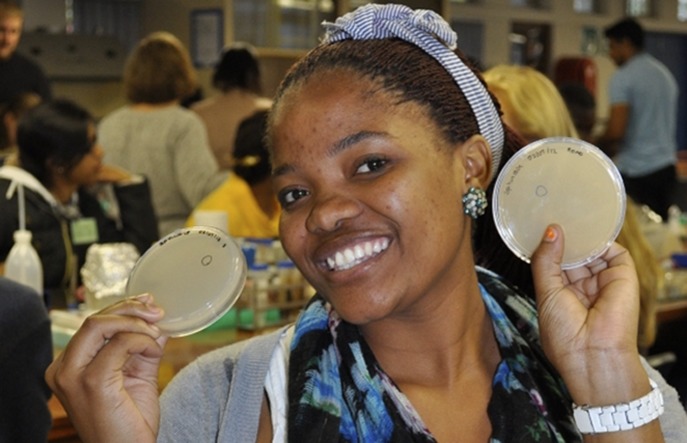
One of the goals of K-RITH is to train new generations of researchers and clinicians in South Africa.

That is why K-RITH will be exploring other approaches, including the development of breath tests, as well as new probes for mRNA or proteins. One of the eight labs will be led by microfluidics expert Frederick Balagaddé, who aims to create improved biomarker, diagnostic, and drug-assessment tools for TB and HIV using microfluidic chips produced in South Africa. Indeed, our larger goal is that these new tools should be invented and developed locally.

The other crucial need is for drugs. No new medicines have been approved since 1967 and successfully treating TB requires at least six months of daily doses of four different drugs. The difficulty of completing the full drug regimen in many patients has led to the increasingly urgent problem of multiple and total drug resistance (Iseman, 2007). Our hope, even expectation, is that the intensive basic research at K-RITH will identify microbiological pathways and mechanisms that will lead to new drug targets. In addition, Jacques Grosset, scientist in residence at K-RITH, will continue his decades of effort to test new drugs in mice, while others will work on new vaccines.

Finally, it is important to remember that K-RITH's mission is not just to conduct basic and translational research. We also want to inspire, train and nurture new generations of South African students, clinicians and researchers. The institute will offer courses, workshops, and masters and PhD programs, in collaboration with UKZN and universities in the United States and Europe. It is also implementing an outreach program to supplement science learning in high schools in the nearby township of Cato Manor. The solution to the urgent global threat of TB is not just a matter of bringing technology and basic science to the heart of an outbreak. It also requires increasing South Africa's own research capabilities and taking advantage of the country's brainpower.

Admittedly, winning the battle against TB and HIV and establishing a world-class research effort are ambitious goals. It might, for instance, be a challenge to sustain K-RITH after the HHMI commitment ends in 2018. But the stark reality is that the current approach to tackling TB has fallen short, so a new approach is needed. If K-RITH stumbles, it will have been a noble experiment, teaching us valuable lessons about the design of future research enterprises. But if it succeeds, as we believe it will, it will offer a new model of science that can be brought to other deadly epidemics around the world.
